# Injectable and Cell-Laden Hydrogel in the Contained Bone Defect Animal Model: A Systematic Review

**DOI:** 10.1007/s13770-023-00569-2

**Published:** 2023-08-10

**Authors:** Chaoxin Wang, Shuyuan Min, Yun Tian

**Affiliations:** 1 Department of Orthopedics, Peking University Third Hospital, Beijing, 100191 China; 2 Engineering Research Center of Bone and Joint Precision Medicine, Ministry of Education, Beijing, 100191 China

**Keywords:** Injectable, Cell-laden, Hydrogel, Bone defects

## Abstract

**Background:**

Due to its high water content and biomimetic properties simulating extracellular matrix (ECM), hydrogels have been used as preferred cell culture and delivery systems. Similarly, cell-loaded hydrogels can be easily injected into target areas in a minimally invasive manner, minimizing surgical trauma, adapting to irregular shaped defects, and benefiting patients. In this study, we systematically reviewed multiple studies on hydrogel-based bone defect research and briefly summarized the progress of injectable and cell-loaded hydrogels in bone defect repair.

**Methods:**

A systematic search was conducted in the PubMed and Web of Science databases using selected search terms.

**Results:**

Initially, 185 articles were retrieved from the databases. After full-text screening based on inclusion and exclusion criteria, 26 articles were included in this systematic review. Data collected from each study included culture model, seed cell type and origin, cell concentration, scaffold material, scaffold shape, experimental animal and site, bioactive agents, and binding method. This injectable and cell-loaded hydrogel shows certain feasibility in bone tissue engineering applications.

**Conclusion:**

Injectable and cell-loaded hydrogels have been widely applied in bone tissue engineering research. The future direction of bone tissue engineering for bone defect treatment involves the use of new hydrogel materials and biochemical stimulation.

## Introduction

In recent years, the increasing occurrence of trauma, tumors, abnormalities, and infections requiring surgical intervention or treatment presents ongoing challenges in the field of orthopedics [1]. Current treatment focus remains on the “gold standard” treatments such as autologous or allogeneic grafts. However, these methods are limited by issues such as limited supply, disease transmission from donor sites, and adverse immune reactions [2, 3]. Consequently, other alternative substitutes are needed nowadays.

Thus, inorganic bone materials like synthetic and metallic substitutes have been applied in the clinic with the advantage of unlimited supply and biocompatibility [4]. However, the incapability of repairing commonly irregular-shape defects and delivering stem cells hamper the wide application of these substitutes.

Although calcium phosphate or polymethyl methacrylate (PMMA) cement can fill irregular-shape defects, the high density and exothermic reactions of bone cement restrict the delivery of regenerative cells and growth factors.

Injectable hydrogels, as a cell carrier increasingly emphasized in tissue engineering and regenerative medicine, may overcome these limitations. Hydrogels have high water content and biomimetic properties simulating the extracellular matrix, making them suitable for preferred cell culture and delivery systems. Additionally, cell-loaded hydrogels can be easily injected into target areas in a minimally invasive manner, adapting to irregular shaped defects, and benefiting patients.

In this study, we systematically reviewed multiple studies on hydrogel-based bone defect research and briefly summarized the progress of injectable and cell-loaded hydrogels in bone defect repair. This review further highlights the content of different cell-loaded hydrogel materials and attempts to answer the following questions: what types of hydrogels have been used? What cells and growth factors have been loaded into hydrogels and have potential applications?

## Materials and methods

### Search strategy

A systematic search was conducted in the PubMed and Web of Science databases using selected search terms. The study was limited to articles written in English.

### Search terms

The following terms included Medical Subject Headings (MeSH) terms and free text phrases: “hydrogel” or “In Situ Hydrogels” or “In Situ Hydrogel” or “Hydrogel, In Situ” or “Patterned Hydrogels” or “Patterned Hydrogel” or “Hydrogel, Patternel”; “bone defect” or “bone loss” or “defect” or “deficiency”; and “injection”. This query aimed to find studies investigating injectable and cell-loaded hydrogels and reporting their potential in bone tissue regeneration.

### Study selection

The entire literature search process was independently performed by two reviewers. Any disagreements were reviewed by a third reviewer. The following inclusion and exclusion criteria were created to determine study eligibility:

#### Inclusion criteria

(1) Studies repairing bone defects using injectable and cell-loaded hydrogels; (2) Original articles written in English only.

#### Exclusion criteria

(1) Human studies only; (2) Reviews, comments, case reports, guidelines, and technical reports; (3) Full texts not available.

### Data extraction

From the included studies, the following study information was recorded: (1) Study characteristics (author, year of publication, and journal name); (2) Intervention details (hydrogel and cells used, growth factors, cross-linking materials and methods, and experimental animals); (3) Mechanical and biological properties.

## Results

**Fig. 1:**
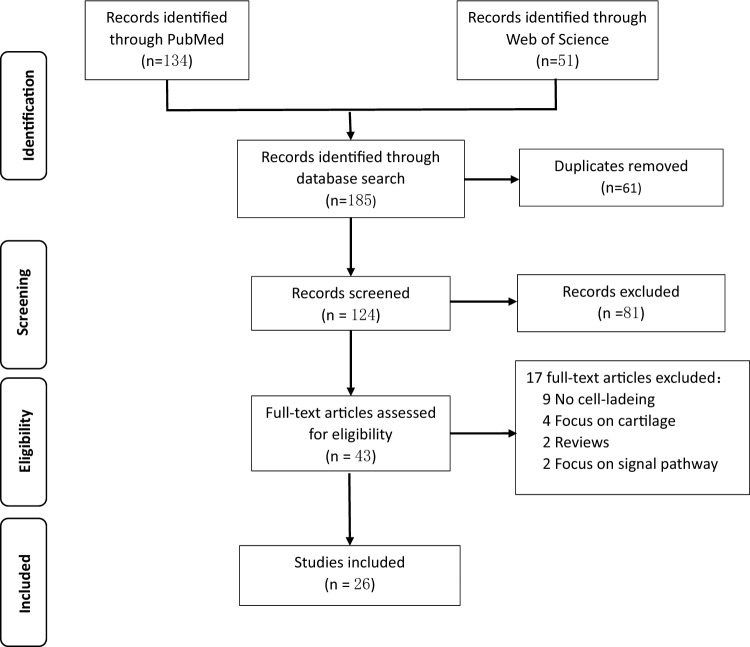
Flow diagram of the systematic literature search

The entire literature search, inclusion, and exclusion process is shown in Fig. 1. Initially, 185 articles were retrieved from the databases. After deduplication (n = 61), 124 articles were screened for their titles and abstracts. As a result, 43 articles were selected for full-text review. The remaining articles included 9 unrelated to stem cells, 4 related to cartilage, 2 were reviews, 2 focused on signaling pathways. Ultimately, this systematic review included 26 articles. Three articles were *in vitro* studies [5–7], three were *in vivo* studies [8–10], and 20 articles were conducted simultaneously [11–29]. Table 1 summarizes the culture model, seed cell type, source, and seeding density. Table 2 summarizes the contents of hydrogels and scaffolds, experimental animal types and defect sites, and defect sizes. Table 3 presents the basic information regarding hydrogel materials, seed cell types, bioactive agents, and binding methods.

**Table 1:** Summary of culture form, seed cell types, sources, and seeding density

Year	Co-culture	Seed cell types	Species	Seeding density(cells/ml)	References
2009	No	MC3T3-E1 preosteoblasts	Mouse	2.4 × 10^7^	[8]
2010	No	BMSC	Goat	NA	[9]
2011	No	BMSC	Rat	1 × 10^7^	[11]
2012	No	ESC	Human	1 × 10^6^	[5]
2014	No	BMSC	Rat	3.3 × 10^5^	[12]
2015	No	BMSC	Rat	1.5 × 10^7^	[13]
2016	No	MC3T3-E1 preosteoblasts	Mouse	1 × 10^6^	[6]
2016	No	BMSC	Rat	1.5 × 10^7^	[14]
2018	No	BMSC	Rat	2 × 10^6^	[10]
2018	No	ADSC	Rabbit	1 × 10^7^	[30]
2018	No	BMSC	Human	NA	[15]
2018	Yes	EC and OB	Mouse	2–5 × 10^6^	[7]
2019	No	BMSC	Ovine	1 × 10^7^	[16]
2020	No	BMSC	Human	2 × 10^7^	[17]
2020	No	BMSC	Rabbit	1 × 10^5^	[18]
2020	No	BMSC	Rat	5 × 10^5^	[19]
2020	No	BMSC	Rat	Single-cell-laden alginate microgels	[20]
2020	No	BMSC	Rat	1 × 10^7^	[21]
2020	No	ADSC	Rabbit	1 × 10^7^	[22]
2020	No	BMSC	Mouse	3 × 10^6^	[23]
2021	No	BMSC	Rat	3.3 × 10^5^	[24]
2021	No	Umbilical cord multipotent MSC	Human	NA	[25]
2021	No	Amnion-derived stem cells	Human	1 × 10^6^	[26]
2021	No	BMSC	Rabbit	NA	[27]
2021	No	BMSC	Rat	1 × 10^7^	[28]
2021	No	BMSC	Rat	1 × 10^6^	[29]

*MSC* mesenchymal stem cell, *BMSC* bone marrow mesenchymal stem cell, *ESC* embryonic stem cell, *ADSC* adipose-derived stem cell, *EC* endothelial cell, *OB* osteoblast

*NA: not available

**Table 2:** Summary of hydrogel contents, experimental animal types, defect sites, and size of defect

Year	Hydrogel contents	Experimental animal	Defect sites	Size of defect	References
2009	GRGDSP-alginate hydrogel	Mouse	back	NA	[8]
2010	modified Pluronic F127 hydrogel	Goat	Tibia	4 holes (Ø 6 mm)	[9]
2011	chitosan/hyaluronic acid/Col hydrogel	Rat	Subcutaneous	NA	[11]
2012	alginate microbeads	NA	NA	NA	[5]
2014	collagen/alginate hydrogel	Rat	Calvarium	2 holes (Ø 5 mm)	[12]
2015	MAEP hydrogel	Rat	Calvarium	1 hole (Ø 8 mm)	[13]
2016	COOH-PLL/laponite	NA	NA	NA	[6]
2016	TGM/PAMAM/GMPs hydrogel	Rat	Calvarium	1 hole (Ø 8 mm)	[14]
2018	Laponite/pNIPAM-co-DMAc/HAPna hydrogel	Rat	Femur	NA	[10]
2018	HA-CPN/PRP/BCP hydrogel	Rabbit	Calvarium	2 holes (Ø 10 mm)	[30]
2018	ALG/hyaluronic acid/BMP-2 hydrogel	Pig	Mandibular bone	1 hole (Ø 10 mm)	[15]
2018	Laponite/gelatin/PCL nanoparticles hydrogel	NA	NA	NA	[7]
2019	RGD-alginate/RGD-hyaluronate/PLG hydrogel	Ovine	Iliac crest	2 holes (Ø 15 mm)	[16]
2020	GelMA/LAP hydrogel	Mouse	Calvarium	1 hole (Ø 5 mm)	[17] [18]
2020	MPEG-PCL-RGD hydrogel	Rabbit	Calvarium	1 hole (Ø 6 mm)	
2020	MC/nHA hydrogel	Rat	Calvarium	2 holes (Ø 6 mm)	[19]
2020	single-cell-laden alginate microgels	Rat	Tibial	2 holes (Ø 1 mm)	[20]
2020	alginate/gelatin/MPs hydrogel	Rat	Femur	2 holes (Ø 2.5 mm)	[21]
2020	nHA/PLGA/chitosan hydrogel	Rabbit	Mandibular bone	1 hole (Ø 8 mm)	[22]
2020	GelMA microsphere hydrogel	Mouse	Distal femurs	1 hole (Ø 1 mm)	[23]
2021	GelMA-Fullerol microspheres	Rat	Calvarium	1 hole (Ø 5 mm)	[24]
2021	dextrin-based hydrogel	Goat	Calvarium	4 holes (Ø 14 mm)	[25]
2021	DBM/OC hydrogels	Rabbit	Tibial	1 hole (Ø 2.5 mm)	[26]
2021	gelatin/ADA/DBM hydrogel	Rabbit	Calvarium	1 hole (Ø 10 mm)	[27]
2021	Alg/Ser/GO hydrogel	Rat	Femur	1 hole (Ø 3 mm)	[28]
2021	GelMA/nHA/SN hydrogel	Rat	Calvarium	1 hole (Ø 8 mm)	[29]

*GRGDSP* the peptide glycine–arginine–glycine–aspartic acid–serine–proline, *MAEP* monoacryloxyethyl phosphate, *COOH*-*PLL* carboxylated poly-l-lysine, *TGM* thermogelling macromer, *PAMAM* polyamidoamine, *GMP* gelatin microparticles, *HA*-*CPN* hyaluronic acid-g-chitosan-g-poly(*n*-isopropylacrylamide), *PRP* platelet-rich plasma, *BCP* biphasic calcium phosphate, *ALG* alginate hydrogel, *BMP*-2 bone morphogenetic protein-2, *PCL* polycaprolactone, *RGD* tripeptide arginine-glycine-aspartic acid, *PLG* poly(lactide-co-glycolide), *MPEG* methoxy polyethylene glycol, *MC* methylcellulose, *nHA* nano-hydroxyapatite, *MPs* magnesium particles, *PLGA* poly(lactic-co-glycolic) acid, *GelMA* gelatin methacryloyl, *DBM* decellularized bone matrix, *OC* oleoyl chitosan, *Ser* sericin, *GO* graphene oxide, *SN* nanosilicat

*NA: not available

**Table 3:** Summary of hydrogel materials, seed cell types, bioactive agents and binding method

Year	Hydrogel materials	Seed cell types	Species	Bioactive agents	Binding method	References
2009	GRGDSP-alginate hydrogel	MC3T3-E1	Mouse	BMP-2	DNA complexed with CaP	[8]
2018	HA-CPN/PRP/BCP hydrogel	ADSC	Rabbit	PRP	Freeform in medium	[30]
2018	ALG/hyaluronic acid/BMP-2 hydrogel	BMSC	Human	BMP-2	Freeform in medium	[15]
2018	Laponite/gelatin/PCL nanoparticles hydrogel	EC and OB	Mouse	VEGF	Freeform in medium	[7]
2020	mGL/LAP hydrogel	BMSC	Human	BMP-2	rAAV	[17]
2020	nHA/PLGAs/chitosan hydrogel	ADSC	Rabbit	VEGF/BMP-2	Freeform in medium	[22]

*VEGF: vascular endothelial growth factor

In these 26 studies, sodium alginate gel was used the most frequently [5, 8, 12, 15, 16, 20, 21, 27, 28], followed by hyaluronic acid (HA) [11, 15, 16, 30] gelatin [7, 21, 27], chitosan [11, 22, 30], and other natural and synthetic polymers. Most articles applied more than one type of hydrogel.

Several studies have investigated the delivery of stem cells via hydrogel microspheres, which offer numerous advantages over conventional bulk hydrogels such as rapid production and size controllability [20, 23, 24]. Various novel functional nanoparticles (NPs) have also been integrated into crosslinkable hydrogel networks to provide desired functionality in some studies [6, 10, 16, 19, 22, 24, 29]. Additionally, bioceramics, including hydroxyapatite, laponite, and β-tricalcium phosphate, have been employed to improve mechanical properties and decrease degradation rates in bone regeneration research. [6, 7, 10, 11, 15, 19, 22, 25, 29, 30].

Of the studies reviewed, two studies used preosteoblasts [6, 8], 23 utilized stem cells for bone differentiation, with MSCs being the most commonly used seeding cell [5, 9–30], while another investigated co-culturing MSCs with endothelial cells [7]. Regarding the source of seed cells, rat/mouse cells were used in 15 studies [6–8, 10–14, 19–21, 23, 24, 28, 29], human cells in five studies [5, 15, 17, 24, 27], rabbit cells in four studies [18, 22, 27, 30], and goat/ovine cells in two studies [9, 16]. Furthermore, the density of seed cells was around 1–240 × 10^5^ cells/ml in most cases.

Localized and sustained introduction of BMPs is used in four studies to promote robust bone tissue formation [8, 15, 17, 22], Vascularization is vital in bone regeneration, and some studies have introduced angiogenic factors into their systems to enhance bone regeneration [7, 22]. Localized gene delivery is an effective and safe alternative for growth factor delivery, as it can be achieved through several methods [7, 15, 22]. And it can be achieved by several ways, including both viral [17] and nonviral [8].

Rats were the most commonly used laboratory animals. As hydrogels do not provide the mechanical robustness required for load-bearing applications, the cranial defect module was used most frequently [7, 12–14, 19, 20, 24, 25, 27, 29, 30], followed by the tibia [9, 20, 26] and femur.

## Discussion

For the therapy of bone defects, the tissue engineering approach is now a popular strategy combining the injectable hydrogel, seed cells, and growth factors for functional reconstruction in a minimally invasive way.

Based on a comprehensive literature review, our research mainly focus on the above three elements of tissue engineering and provide potential guidelines for future studies and the clinical application of stem cells and biomaterial-based bone regeneration.

### Hydrogels for reconstruction of bone defects

#### Alginate hydrogels and their derivatives

Alginate is a naturally-extracted polysaccharide from brown algae containing glucuronic and mannuronic acids, which make it more selective for binding to Ca^2+^ ions to form hydrogel. It has been applied in numerous tissue engineering experiments due to its favorable properties including biocompatibility, biodegradability, and facile gelation process. Furthermore, their ability to encapsulate MSCs or bioactive components suggested that this hydrogel is a promising suitable injectable polymer for controlled delivery and bone tissue engineering. ChuanfengAn etc. employed alginate hydrogel beads for microencapsulation of MSCs to support cell proliferation and osteoblastic differentiation [31]. However, alginate hydrogel has many limitations for tissue engineering. First, its poor mechanical properties and short-term stability do not ensure maintenance of the regenerated tissue. Second, its poor cell adhesion properties provide limited support for cellular functionality. For this reason, alginate is often used in a chemically modified form. Ganesh C. Ingavle etc. modified the alginate with the RGD peptide sequence as adhesion ligands that can promote cell attachment, proliferation, and bone formation [16].

#### Chitosan hydrogels and their derivatives

Chitosan is an amino polysaccharide derived from crab and shrimp shells, composed of repeating units of glucosamine and *N*-acetylglucosamine linked by β − (1–4) glycosidic bonds. Due to its low immunogenicity, chitosan-based hydrogels have shown significant potential for promoting cell proliferation and adhesion, and have displayed great potential for bone tissue regeneration [16]. However, the mechanical strength, degradation, and osteogenic activity of pure chitosan are poor, which can affect the final repair effect. To improve its mechanical performance and biological activity, chitosan-based hydrogels need to be combined with other synthetic or natural polymers and bioactive molecules to construct multifunctional biomaterials.

#### Hyaluronic acid hydrogels and their derivatives

Hyaluronic acid is a copolymer of D-glucuronic acid and *N*-acetyl-D-glucosamine, which is present in all tissues. The biodegradability, cell adhesion, migration, proliferation, and differentiation properties of hyaluronic acid are important for its potential use as a tissue engineering construct [32]. Moreover, because hyaluronic acid can be enzymatically degraded, it does not cause an immune reaction and can serve as a carrier for injection. *In vivo*, hyaluronic acid can promote the construction of a matrix around cells, providing a suitable microenvironment for stem cell differentiation. Additionally, hyaluronic acid hydrogels are commonly used as carriers for growth factors, such as BMPs, to promote osteogenesis. However, the mechanical performance of hyaluronic acid hydrogels is poor. To overcome these drawbacks, a range of modification methods has been developed. Chemical modification of hyaluronic acid can be achieved by reacting its carboxylic groups with various hydroxyl or amine-containing groups to form derivatives with better biocompatibility and controllable degradation [32].

#### Gelatin hydrogels and their derivatives

Gelatin is a hydrolysis product extracted from collagen, which is the main component of cartilage tissue extracellular matrix (ECM). Gelatin has excellent cell adhesion, biocompatibility, and biodegradability properties. However, it has a drawback in that its physical crosslinking stability is low *in vivo*. Therefore, chemical modification of gelatin is required before use in gelatin hydrogels [31]. GelMA retains most of the functional amino acid groups of gelatin, thus possessing excellent cell adhesion properties. Due to its inherent biological activity and tunable physicochemical properties, GelMA has been widely used in tissue engineering applications. Although these hydrogels are easy to adapt to microfluidic technology [33], they also have some drawbacks. The free radicals released during photodependent crosslinking, residual monomers and photoinitiators in hydrogel microparticles have high cell toxicity [34]. Moreover, the crosslinking strength cannot be controlled during this process, inadequate crosslinking may lead to microsphere fusion and rupture, while excessive crosslinking will greatly reduce the internal porosity of the microspheres, affecting cell behavior. Finally, excessive ultraviolet irradiation during the crosslinking process may reduce cell survival.

#### Typical synthetic (composite) polymer-based hydrogels

The main drawbacks of natural hydrogels include poor mechanical properties, fast and unpredictable degradation, and strong dependence on the individual, enzyme levels, and injection site. Chemical modifications of hydrogels, such as the addition of specific functional groups and macromolecules, can overcome their inherent limitations by improving their mechanical properties and adjusting their biodegradability [35]. Synthetic hydrogels have great potential for use in regenerative medicine due to their programmable and reproducible properties [36]. Poly(ethylene glycol) (PEG), poly(2-hydroxyethyl methacrylate) (PHEMA), and polyvinyl alcohol (PVA) are the most commonly used hydrogels in the biomedical field because of their high hydrophilicity, non-toxicity, and ease of functionalization through chemical reactions. However, most hydrogels are usually weak and easily degraded under physiological conditions [37]. To overcome this drawback, a special class of interpenetrating network (IPN) polymers, called double network (DN) hydrogels, has recently been synthesized to address issues such as rapid crosslinking, injectability, and cell compatibility.

### Various types of cells encapsulated in hydrogels

#### Preosteoblasts and osteoblasts

Pre-osteoblasts are good cell sources for bone tissue engineering. Although stem cells have self-renewal and pluripotent differentiation abilities, making them a better source. Pre-osteoblasts have stronger osteogenic properties than MSCs, so they still have good research and application prospects.

#### Stem cells

MSCs are important tools in regenerative medicine due to their chemotaxis, multidirectional differentiation, and immunomodulatory capabilities [38]. In recent years, MSCs including BMSCs, ADSCs, and hESCs have been widely studied. They all have some osteogenic ability in hydrogels both *in vitro* and *in vivo*.

BMSCs are a cell source for many bone tissue engineering applications because they have higher proliferation capacity and are generally easier to obtain than mature osteoblasts [39].

ADSCs are widely used as seed cells for tissue engineering due to their ease of acquisition, strong proliferative activity, multipotent potential, and immunomodulatory capability [40].hESCs are also a very promising source of cells because they have long-term proliferation and self-renewal abilities and can differentiate into almost all cell types [41].

#### Co-culture of endothelial cells and stem cells

Bone is a highly vascularized tissue, and osteogenesis and angiogenesis are coupled. There is a synergistic effect between bone cells and endothelial cells during bone regeneration. When MSCs and endothelial cells are co-cultured, the markers for osteogenesis and blood vessel generation are enhanced compared to single culture of the cells. On the other hand, vascular injury can inhibit bone growth and induce skeletal diseases [42]. In the construction of synthetic biomaterials for bone regeneration, insufficient formation of a vascular network can affect bone healing and even lead to tissue necrosis. However, developing vascularized bone implants remains a challenge until now.

### Growth factors on hydrogels

In the natural process of bone defect repair, MSCs and many other types of cells interact with growth factors, among which bone morphogenetic protein-2 (BMP-2) plays a dominant role in promoting bone defect healing due to its strong osteogenic properties. Vascular endothelial growth factor (VEGF) is the most important growth factor for promoting angiogenesis and new bone formation *in vivo* [43].

#### BMP-2

BMP-2 is one of the BMPs that have been shown to have strong osteoinductive effects [44]. The molecule is involved in the osteoinductive signaling pathway, promoting differentiation of MSCs into osteoblasts. BMP-2 has great potential in bone regeneration, but a large amount of protein is required to produce an effect due to the degradability of proteins *in vivo* [45].

#### VEGF

Vascularization is a key process in bone regeneration and a limiting step in the healing of large area bone defects [46, 47]. In the past decade, hydrogels have been functionalized by loading biologically active molecules into drug delivery systems, forcing them to locally deliver at the required time in sufficient doses. Among them, vascular endothelial growth factor can maintain long-term stability and half-life with few adverse reactions. In this case, hydrogels wrapped with these biologically active molecules can regulate and promote cell differentiation, proliferation, migration, and vascularization.

## Conclusion

Based on the literature review, cell-laden injectable hydrogels have been widely applied in bone tissue engineering research. Cell-laden hydrogels have demonstrated excellent injectability, cell viability, and osteogenic properties in both *in vivo* and *in vitro* experiments. However, most studies have not analyzed the mechanical properties of the regenerated bone tissue, and the newly-formed bone may not possess satisfactory mechanical performance, limiting the application of injectable hydrogels in weight-bearing bone defects. Moreover, fewer studies have focused on reconstructing a favorable microenvironment with more M2 macrophages and less inflammation, which is also a key factor in promoting bone regeneration. Therefore, the future direction of bone tissue engineering involves the use of novel hydrogel materials combined with biochemical and biomechanical stimuli to ensure that the regenerated bone tissue is well reshaped into natural bone.
